# Feasibility and Possible Effects of Mindful Walking and Moderate Walking in Breast Cancer Survivors: A Randomized Controlled Pilot Study With a Nested Qualitative Study Part

**DOI:** 10.1177/15347354211066067

**Published:** 2022-01-19

**Authors:** Maren Luise Schröder, Barbara Stöckigt, Sylvia Binting, Tatjana Tissen-Diabaté, Nikola Bangemann, Ute Goerling, Matthias Kröz, Jens-Uwe Blohmer, Miriam Ortiz, Benno Brinkhaus

**Affiliations:** 1 Charité – Universitätsmedizin Berlin, Berlin, Germany; 2 Carl-Thiem-Klinikum Cottbus, Cottbus, Brandenburg, Germany; 3 Research Institute Havelhoehe, Berlin, Germany; 4 University Witten/Herdecke, Witten, Germany; 5 Hospital Arlesheim, Switzerland

**Keywords:** breast cancer, walking, mindfulness, complementary medicine, mixed-methods, pilot study, MBSR

## Abstract

**Introduction:**

Breast cancer (BC) survivors often suffer from disease- and therapy-related long-term side-effects. The study aim was to explore the feasibility, adherence, and individual experiences as well as possible effects of 2 different walking interventions in BC patients.

**Methods:**

This randomized controlled, pragmatic pilot trial included a qualitative study component. BC patients were randomized to either mindful walking (MFW) with mindfulness exercises and walking or moderate walking (MW) alone in weekly group sessions over 8 weeks. After 8 and 16 weeks, satisfaction, and self-perceived effectiveness as well as different health-related outcomes including health-related (WHOQOL-BREF) and disease-specific quality of life (FACT-G), perceived stress (PSQ) and cancer-related fatigue (CFS-D) were assessed. ANCOVA was used to evaluate differences in study outcomes. Qualitative data included 4 focus group interviews including 20 patients and were analyzed using a directed qualitative content analysis approach.

**Results:**

Altogether, 51 women (mean age 55.8 years (SD 10.9)) were randomized (n = 24 MFW; n = 27 MW). Both groups would recommend the course to other BC patients (MFW 88.9%; MW 95.2%) and showed possible improvements from baseline to week 8, without statistically significant difference between groups: WHOQOL-BREF (MFW: adjusted mean 65.4 (95% confidence interval (CI), 57.1-73.7); MW: 61.6 (53.6-69.6)); FACT-G (MFW: 76.0 (71.5-80.5); MW: 73.0 (68.5-77.4)); PSQ (MFW: 45.3 (40.5-50.1); MW: 45.4 (40.8-50.0)); CFS-D (MFW: 24.3 (20.8-27.8); MW: 25.5 (22.1-28.8)). Improvements lasted until the 16-weeks follow-up. The qualitative analysis suggested that MFW primarily promoted mindfulness, self-care, and acceptability in BC patients, whereas MW activated and empowered the patients as a result of the physical exercise.

**Conclusion:**

Both study interventions were positively evaluated by patients and showed possible pre-post effects in disease-specific health-related outcomes without differences between groups. The qualitative analysis results indicate that different resources and coping strategies were addressed by the 2 study interventions.

**Trial registration:**

DKRS00011521; prospectively registered 21.12.2016; https://www.drks.de/drks_web/navigate.do?navigationId=trial.HTML&TRIAL_ID=DRKS00011521.

## Introduction

Breast cancer (BC) is the most commonly diagnosed cancer among women.^1,2^ Although the incidence has increased, the mortality rate has decreased due to improved early detection measures and enhanced therapies.^1^ BC survivors often suffer from diagnosis- and therapy-related long-term consequences,^3^ such as reduced health-related quality of life,^4,5^ distress,^6^ anxiety and depression,^7-9^ cancer-related fatigue,^6,10,11^ insomnia,^12^ reduced capacity and pain.^13^ Therefore, the consideration and treatment of long-term side-effects in BC survivors have become increasingly important.^1^

Scientific evidence by reviews and meta-analyses in BC patients has shown that physical activity has positive effects on health-related quality of life^3,14-16^ as well as in reducing the risk of recurrence.^17,18^ Further clinical studies have shown beneficial effects of physical activity in BC patients regarding cancer-related fatigue,^19^ anxiety and depression.^15^ Walking in particular has shown positive effects in reducing fatigue symptoms during^20,21^ and after primary oncologic treatment^22^ as well as in improving health-related quality of life and well-being.^22^ In contrast, in a randomized controlled trial, walking was not effective at reducing anxiety and depression during chemotherapy.^20^

Mindfulness can be defined as directing attention to the present moment, including all internal and external thoughts, feelings, and bodily sensations, and encountering those with openness, curiosity, and acceptance without valuating them.^23,24^ Clinical studies in BC patients have revealed positive effects of Mindfulness-Based Stress Reduction Interventions (MBSR) on stress reduction,^25-27^ improving health-related quality of life,^27,28^ and alleviating anxiety and depression^25,27,29^ as well as cancer-related fatigue.^26,27,29^ In systematic reviews and meta-analyses, MBSR was positively evaluated and recommended for BC patients.^30-32^

We developed a combination of walking and mind-body medicine techniques, that addresses physical exercise through walking as well as stress reduction and relaxation through mindfulness meditation. The intervention was proved successfully in psychologically distressed individuals,^33^ however the effects of a mindful walking intervention have not yet been established for BC patients.

The aim of this study was to explore the feasibility, adherence, and individual experiences of the combination of walking and mind-body techniques (MFW) compared to moderate walking alone (MW) as well as possible beneficial effects of the MFW intervention compared to MW in BC patients after primary treatment. Disease-specific health-related outcomes were triangulated with qualitative data.

## Methods

### Design

A randomized controlled, two-armed, pragmatic, single-center pilot interventional trial including a qualitative study component (mixed-methods approach) was performed at the Institute for Social Medicine, Epidemiology and Health Economics, Charité – Universitätsmedizin Berlin, Germany. Within the mixed-methods approach quantitative and qualitative data were triangulated to gain a more comprehensive understanding of the subject.

BC patients who had finished their primary oncologic treatment for at least 6 months were randomized to a group intervention program of either mindful walking (MFW) or moderate walking without mind-body medicine techniques (MW). The intervention phase of either MFW or MW lasted 8 weeks, followed by an 8-week follow-up period without intervention. Outcomes were assessed at baseline and after 4, 8, and 16 weeks. Qualitative data were collected after the follow-up period of 16 weeks. In the conception phase of the study, several stakeholder meetings took place with a patient advocate from a BC support group, gynecologic oncologists, psycho-oncologists, physicians specialized in integrative medicine, scientists, and mindfulness meditation trainers. They were involved in the selection of outcome parameters and inclusion and exclusion criteria. Furthermore, a trial run of the MFW intervention program with 5 BC patient volunteers was performed.

Patients were randomized in one study center to either MFW or MW using a 1:1 allocation ratio. The randomization list was generated with SAS Version 9.4. (SAS Institute, Cary, NC, USA). A stratified block randomization with variable block length was performed. Randomization was stratified by past chemotherapy due to BC and current use of antihormonal therapy. After patients were included in the study, the study physician informed the blinded study nurse via telephone about the participant’s number, which was assigned subsequently. The study secretary, who had access to the computer-generated randomization list, sent the information about the allocation to study group back to the study physician via fax. The study physician informed the patient about the allocated study group, after the patient had filled out the first questionnaire.

The study was approved by the local ethics review board and yielded a positive vote (reference number: EA1/201/16; 06.07.2016) based on the Declaration of Helsinki and ICH E6 Guideline for Good Clinical Practice (GCP). Amendments were submitted and approved in July 2016, October 2016, and July 2017. Written informed consent was obtained from all individual participants included in the study.

### Participants and Setting

Participants were recruited at the Outpatient Clinic for Integrative Medicine, the Breast Center, and the Comprehensive Cancer Center of the Charité – Universitätsmedizin Berlin as well as at cooperating oncological and gynecological practices in Berlin and via subway advertisement. Prior to study inclusion, a telephone screening and a subsequent personal screening examination were carried out by study personnel. The following inclusion criteria were defined: female BC patients, ≥18 years of age, completion of the primary cancer therapy (operation, chemotherapy, radiation therapy) for at least 6 months before the beginning of the study intervention and increased levels of stress (>40 mm) on a visual analog scale (VAS 0-100 mm). The following exclusion criteria were defined: non-regional metastases, self-described limited walking ability, regular meditation or walking practice for more than 60 minutes or more than once a week, an upcoming rehabilitation or meditation course within the next 16 weeks and clinically relevant and restricting cardiac disease, pulmonary disease, organic and/or mental disease.

### Study Interventions

Participants of MFW attended a 90-minute mindful walking group session once a week for 8 weeks under the guidance of qualified and experienced mindfulness and meditation trainers. The number of participants was limited to a maximum of 10. Various meditation and mindfulness exercises in combination with short walking practices (“good-mood-walking”) were performed: breathing meditation, body scan, Metta meditation and walking meditation. The participants received handouts and audio files with content and exercises of the group sessions for home practice.

Participants of MW attended a 90-minute outside walking group session once a week for 8 weeks under the guidance of a certified walking trainer. In a group of a maximum of 10 participants, moderate walking was practiced at a speed of approximately 4 to 5 km/hour adapted to the group. The length of the walking route was 5.5 km. No walking poles were used. Adherence to the correct walking technique was guided and supervised by the trainer. In addition to the mere walking distance, initial warming-up and final stretching exercises were performed. There were no mindfulness or meditation exercises carried out in this group. All participants received a handout with the walking route and course dates.

Both groups were regularly encouraged to practice the interventions as often as possible at home.

### Outcome Measurements

Socio-demographic and disease-specific information was collected at baseline as part of the personal screening examination. Patient satisfaction, feasibility, and self-perceived effectiveness were recorded by self-developed questions; adverse events (AE), number of home practice times and personal motivation to practice were documented weekly in an exercise protocol. AEs were systematically assessed by the patients’ weekly diary during the 8 weeks of intervention. If AEs were reported consistently by 1 person, they were counted every time they were mentioned. All AEs were categorized based on the CTCAE-Guideline from 2017, Version 5.^34^

In addition, disease-specific health-related outcomes were assessed using standardized and validated questionnaires in German language. Quality of life was measured generally by the *World Health* Organization *Quality of Life Assessment* (WHOQOL-BREF), a short version of the WHOQOL-100, with 26 questions clustered in 4 domains (physical, psychological, social relationships, and environment) and answered on a 5-point Likert scale.^35,36^ The minimal clinically important difference (MCID) in the WHOQOL-BREF is described in patients with advanced lung cancer based on distribution-based methods for a 0.5 standard deviation as approximately 8.8% in the general facet.^37^ Disease-specific quality of life was measured by the Functional Assessment of Cancer Therapy: General (FACT-G) consisting of 27 items clustered in 4 domains (physical, social/family, emotional, and functional well-being) and answered on a scale from 0 to 4 .^38,39^ The MCID in the FACT-G total score is described as a range from 4 to 10 points, related to either improvement or worsening.^40-42^ Based on a combination of an anchor- and distribution-based approach,^40^ an improvement of ≥6 points was considered a MCID in this study. Stress experience was measured by the short version of the Perceived Stress Questionnaire (PSQ—short version) containing 20 items regarding 4 domains (worries, tension, joy, and demands) answered on a 4-point Likert scale.^43,44^ Cancer-related fatigue was measured by the Cancer Fatigue Scale (CFS-D) containing 15 questions on physical, affective and cognitive fatigue answered on a 0- to 4-point scale.^45^ Based on general findings by Osoba et al^46^ for the EORTC QLQ C30, whereby a clinically relevant change occurs between 5% and 10%, a clinically relevant CFS-D score would be 3 to 6 points. This has not yet been evaluated separately for the CFS-D. Anxiety and depression were measured by the Hospital Anxiety and Depression Scale (HADS), which includes 14 items divided in 2 subscales (anxiety and depression) of 7 items each, on a 0- to 3-point scale.^47-51^ Further measured outcomes include the General Self-Efficacy Short Scale (ASKU) with 3 items regarding subjective expectations of competence,^52^ the Freiburg Mindfulness Inventory (FFA—short form) with 14 items relating to mindfulness,^53,54^ the Trait Autonomic Regulation with 18 questions regarding self-perceived autonomic regulations (T-HKF) in 3 subscales (orthostatic-circulatory, rest/activity, and digestive regulation),^55^ and pain on a numeric rating scale from 1 to 10 (NRS 1-10).

### Statistics

Due to the exploratory nature of the study, no sample size planning was done prior to the study. For pragmatic reasons, a target size of at least 50 patients was selected as this seemed to be a realistic number for this single-center study. The statistical analyses of socio-demographic and disease-specific baseline data and collected outcome parameters were carried out descriptively. Data analyses followed a predefined statistical analysis plan (SAP). Analysis of data was carried out with R (Version 3.6).^56^

Continuous endpoints were analyzed by an analysis of covariance (ANCOVA). Explanatory variables in this model were the intervention group (MFW/MW), stratification variables (chemotherapy in the past due to BC/current use of antihormonal therapy) and the respective baseline value. Results of the estimated intervention effect were presented as the adjusted means per intervention group, with 95% confidence intervals and two-sided *P*-values. As part of the statistical evaluation, the interpretation of the results was purely exploratory. Due to its exploratory character, no primary outcome parameters were defined in this study. The analyses were performed for the full analysis set (FAS) following an intention-to-treat-principle. Missing values were not replaced.

### Nested Qualitative Study

For the qualitative study component, all patients who participated in at least 6 of 8 course dates were contacted after the follow-up period and asked if they were interested in participating in a focus group. Four semi-structured focus group interviews (2 per study arm) were conducted after the follow-up period by M.S. and B.S., 2 experienced qualitative researchers. Each focus group interview consisted of 4 to 6 participants. Based on a predefined interview guideline, the focus of the interviews was the subjective perceived experience and the impact of both study interventions on the course of the disease, on everyday life and experiences and the subjective perceived effects, feasibility, and adherence. Audio-recorded focus group interviews were transcribed verbatim and pseudonymized. The subsequent data analysis was performed using MAXQDA^®^. The interview material was analyzed deductively and inductively using a directed qualitative content analysis approach.^57^ Qualitative content analysis is a popular and widely used method in health research to analyze text data. The direct approach is characterized by the fact that existing theoretical frameworks can be upheld and extended conceptually.^57^ The results of the analysis were discussed regularly by the research team and in an interdisciplinary qualitative working group (Qualitative Research Network, Charité – Universitätsmedizin Berlin) to ensure intersubjectivity.

## Results

**Figure 1.**
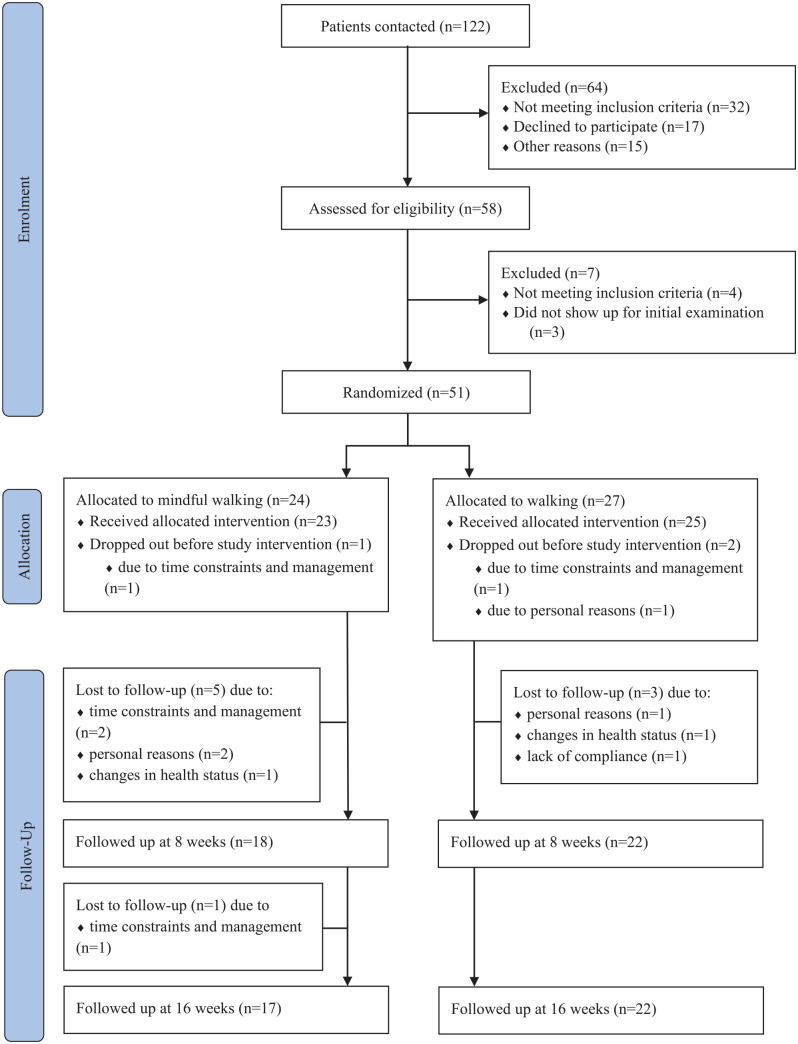
Flow chart.

Between September 2017 and November 2018, 122 BC patients were screened for eligibility; 51 of those patients were randomized into groups (MFW n = 24; MW n = 27) (Figure 1). During the study, 12 participants (MFW n = 7; MW n = 5) withdrew from participation, of whom 3 participants (MFW n = 1; MW n = 2) withdrew before the study intervention. Reasons for withdrawal before and during the study intervention were time constraints and management (n = 5), personal reasons (n = 4), changes in health status (n = 2), and lack of compliance (n = 1).

### Baseline Characteristics

The average age of patients included in the study was 55.8 years (SD 10.9), with a mean body mass index (BMI) of 25.2 (SD 3.8) (Table 1). Differences between groups were found in the status of menopause (58.3% of the MFW and 81.5% of the MW participants were postmenopausal) and mean time since the end of primary therapy (MFW 3.5 years (SD 2.9; median 2.7); MW 4.8 years (SD 5.8; median 1.9)). Regarding diagnosis- and therapy-related side-effects of BC, the average self-perceived functional capacity (%) of all women was 65.5 (SD 16.0). The average level of perceived distress (VAS, 0-100 mm) was 67.0 mm (SD 2.8). Overall, 96.1% of all women had lasting diagnosis- and therapy-related side-effects, such as fatigue symptoms, neuropathy, arthralgia, hot flushes, insomnia, and lymphedema. Outcome parameters at baseline were comparable between groups (Table 2). Of all randomized patients, 63% participated in 6 of 8 course dates.

**Table 1.** Baseline Characteristics.

Characteristics	All participants	Mindful walking	Walking
	n = 51	n = 24	n = 27
	Mean ± SD/n (%)	Mean ± SD/n (%)	Mean ± SD/n (%)
Age (years)	55.8 ± 10.9	55.4 ± 13.0	56.2 ± 8.8
Body Mass Index (kg/m²)	25.2 ± 3.8	25.0 ± 3.4	25.4 ± 4.2
Pre-/Perimenopausal	15 (29.4)	10 (41.7)	5 (18.5)
Postmenopausal	36 (70.6)	14 (58.3)	22 (81.5)
Time since the end of primary therapy (years)	4.2 ± 4.6	3.5 ± 2.9	4.8 ± 5.8
- median (years)	2.5	2.7	1.9
Radiotherapy	41 (80.4)	18 (75.0)	23 (85.2)
Chemotherapy	25 (49.0)	12 (50.0)	13 (48.2)
Antihormonal therapy	37 (72.6)	16 (66.7)	21 (77.8)
Continuing	31 (83.8)	14 (87.5)	17 (81.0)
Completed	6 (16.2)	2 (12.5)	4 (19.1)
Antibody therapy	8 (15.7)	5 (20.8)	3 (11.1)
Continuing	2 (25.0)	1 (20.0)	1 (33.3)
Completed	6 (75.0)	4 (80.0)	2 (66.7)
Persisting side-effects through therapy	49 (96.1)	23 (95.8)	26 (96.3)
Functional capacity (%)	65.5 ± 16.0	65.1 ± 14.9	65.8 ± 17.3
Stress VAS (0-100 mm)	67.0 ± 2.8	68.9 ± 13.7	65.3 ± 11.9

Values are shown as the absolute numbers (n) and percentages (%), means and standard deviations (SD) or medians.

Abbreviation: VAS, visual analog scale.

**Table 2.** Outcome Parameters at Baseline.

Outcome parameter	All participants	Mindful walking	Walking
	n = 51	n = 24	n = 27
	Mean ± SD	Mean ± SD	Mean ± SD
WHOQOL-BREF^†^			
Physical domain (0-100)^†^	59.9 ± 15.6	57.9 ± 16.4	61.7 ± 14.9
Psychological domain (0-100)^†^	55.4 ± 13.1	54.3 ± 16.1	56.4 ± 9.9
Social relationships domain (0-100)^†^	61.0 ± 18.9	62.7 ± 20.9	59.6 ± 17.1
Environment domain (0-100)^†^	70.6 ± 12.4	69.8 ± 13.7	71.4 ± 11.3
Overall perception of life and health (Question 1 + 2) (0-100)^†^	53.4 ± 16.2	52.6 ± 18.4	54.2 ± 14.3
FACT-G^†^			
Total score (0-108)^†^	66.8 ± 15.0	64.4 ± 18.4	69.1 ± 10.8
Physical well-being (0-28)^†^	18.4 ± 5.0	17.9 ± 5.9	18.8 ± 4.2
Social/Family well-being (0-28)^†^	17.7 ± 5.2	17.2 ± 5.9	18.2 ± 4.5
Emotional well-being (0-24)^†^	15.3 ± 4.5	14.5 ± 5.2	16.0 ± 3.6
Functional well-being (0-28)^†^	15.3 ± 5.0	14.8 ± 6.0	15.7 ± 3.9
Cancer Fatigue Scale (CFS-D)^‡^			
Total score (0-60)^‡^	30.3 ± 8.1	30.0 ± 9.6	30.4 ± 6.8
Physical fatigue subscale (0-24)^‡^	14.0 ± 4.2	13.8 ± 5.1	14.2 ± 3.2
Affective fatigue subscale (0-20)^‡^	5.9 ± 2.1	6.0 ± 2.5	5.9 ± 1.7
Cognitive fatigue subscale (0-16)^‡^	10.3 ± 3.6	10.2 ± 3.6	10.4 ± 3.7
Perceived Stress Questionnaire (PSQ—short form)			
Overall score (0-100)⁺	54.6 ± 15.7	53.6 ± 18.8	55.4 ± 12.7
Worries subscale (0-100)⁺	48.3 ± 22.9	48.9 ± 28.3	47.7 ± 17.4
Tension subscale (0-100)⁺	60.7 ± 19.4	61.7 ± 22.5	59.8 ± 16.5
Joy subscale (0-100)⁺	39.8 ± 19.4	44.4 ± 23.1	35.7 ± 14.6
Demands subscale (0-100)⁺	49.1 ± 15.4	48.3 ± 17.2	49.8 ± 13.9
Hospital Anxiety and Depression Scale (HADS)′			
Total score (0-42)′	15.8 ± 7.3	16.9 ± 8.4	14.9 ± 6.2
Anxiety subscale (0-21)′	9.2 ± 4.1	9.8 ± 4.7	8.7 ± 3.4
Depression subscale (0-21)′	6.6 ± 4.0	7.1 ± 4.1	6.1 ± 3.8
Trait autonomic regulation^#^			
Autonomic regulation total score (18-54)^#^	37.8 ± 5.4	37.0 ± 5.1	38.6 ± 5.7
Orthostatic-circulative aR subscale (7-21)^#^	15.5 ± 2.9	14.8 ± 2.9	16.1 ± 2.9
Rest-/activity aR subscale (8-24)^#^	15.2 ± 3.3	15.0 ± 3.2	15.4 ± 3.5
Digestive aR subscale (3-9)^#^	7.2 ± 1.5	7.2 ± 1.3	7.1 ± 1.7
General Self-efficacy short scale (ASKU) (0-5)″	3.7 ± 0.7	3.7 ± 0.7	3.6 ± 0.7
Freiburg Mindfulness Inventory (FFA) (14-56)^**^	32.9 ± 7.1	33.9 ± 8.8	32.0 ± 5.1
Pain (NRS) (0-10) ^##^			
because of cancer	3.1 ± 2.6	3.5 ± 2.9	2.7 ± 2.3
because of other disease	3.7 ± 2.5	4.0 ± 2.6	3.5 ± 2.5

Values are shown as the means and standard deviations (SD).

Abbreviations: WHOQOL-BREF, World Health Organization Quality of Life Assessment; FACT-G, functional assessment of cancer therapy-general; aR, autonomic regulation; NRS, numeric rating scale.

† Higher values indicate better quality of life.

‡ Lower values indicate less suffering of fatigue.

⁺ High overall score indicates high level of perceived stress.

′ Higher values indicate higher severity of symptoms.

# Lower values indicate less autonomic regulation.

″ Higher values indicate better self-efficacy.

** Higher values indicate higher mindfulness.

## Lower values indicate less suffering of pain.

### Adherence to Intervention, Feasibility, and Self-Perceived Effects

Exercise motivation, recorded weekly in an exercise protocol, was relatively constant over 8 weeks: overall, 75% were highly motivated (MFW 6%; MW 10%) or motivated (MFW 71%; MW 64%). The mean individual home practice frequency over 8 weeks, recorded weekly in an exercise protocol, differed between groups in favor of MFW: 7% of MFW participants did not practice, in contrast to 35% of MW participants; 80% of MFW participants practiced some days of the week, respectively 64% of MW participants; 13% of MFW participants practiced all days. respectively 1% of MW participants. Most patients were very satisfied (MFW 44.4%; MW 66.7%) or satisfied (MFW 55.6%; MW 23.8%) with the study intervention, perceived the study intervention as very effective (MFW 22.2%; MW 19.1%) or effective (MFW 72.2%; MW 71.4%) and would therefore recommend the course to other BC patients (MFW 88.9%; MW 95.2%).

### Adverse Events

No severe AEs occurred during the study period. All AEs could be classified as grade 1 (mild).^34^ Related to the study intervention, 47 AEs (MFW 23; MW 24) were documented by n = 15 patients (MFW 8; MW 7): 3 patients (MFW n = 1; MW n = 2) reported 13 incidents of back pain; 8 patients (MFW n = 4; MW n = 4) reported 19 incidents of other musculoskeletal and connective tissue disorders; 3 patients (MFW n = 2; MW n = 1) reported 6 incidents of skin and subcutaneous tissue disorders; 4 patients (MFW) reported 7 incidents of vascular disorders like dizziness or hypertension; and 1 patient (MFW) reported 2 incidents of a psychiatric disorder during meditation practice (panic attack).

### Outcome Parameters at 8 and 16 weeks

**Figure 2.**
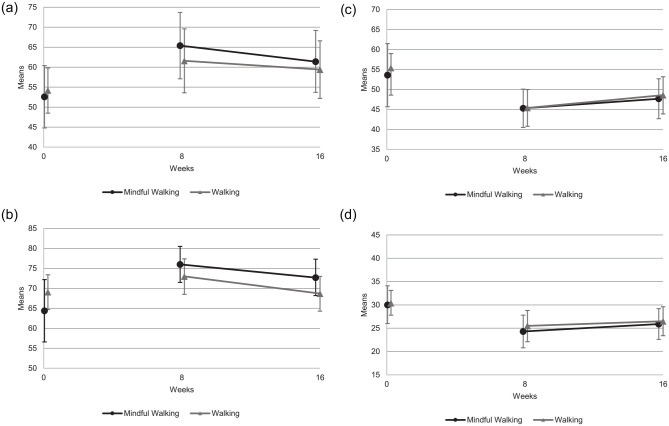
Outcome parameters over 16 weeks. Baseline values are not adjusted means with 95% CI. Outcome parameters at 8 and 16 weeks are means adjusted for baseline value and stratification variables with 95% CI. (a) WHOQOL-BREF overall perception of life and health (0-100)^†^ over 16 weeks. ^†^Higher values indicate better quality of life. (b) FACT-G total score (0-108)^†^ over 16 weeks. ^†^Higher values indicate better quality of life. (c) PSQ (short form) overall score (0-100)^+^ over 16 weeks. ^+^High overall score indicates high level of perceived stress. (d) CFS-D total score (0-60)^‡^ over 16 weeks. ^‡^Lower values indicate less suffering of fatigue.

Both groups improved substantially from baseline to week 4 (Supplemental File 1), 8 and 16, with no statistically significant differences between groups after the interventions at 8 weeks for WHOQOL-BREF overall perception of life and health (MFW: adjusted mean 65.4 (95% CI, 57.1-73.7); MW: 61.6 (53.6-69.6) (*P* = .491)), FACT-G total score (MFW: 76.0 (71.5-80.5); MW: 73.0 (68.5-77.4) (*P* = .328)), PSQ overall score (MFW: 45.3 (40.5-50.1); MW: 45.4 (40.8-50.0) (*P* = .972)) or CFS-D total score (MFW: 24.3 (20.8-27.8); MW: 25.5 (22.1-28.8) (*P* = .630)) (Table 3). Findings were similar for all other outcome parameters (Supplemental File 2) and subscales, except for WHOQOL-BREF subscale social relationships (*P* = .044) and FACT-G subscale social/family well-being (*P* = .019) after 16 weeks. Both subscales showed a statistically and clinically relevant group difference in favor of MFW. Both groups showed possible positive effects compared to baseline after the intervention period at 8 weeks, and these improvements lasted until the 16-weeks follow-up (Figure 2a–d).

**Table 3.** Selected Outcome Parameters at 8 and 16 weeks.

Outcomes	8 weeks			16 weeks		
	Mindful walking	Walking	*P*-value	Mindful walking	Walking	*P*-value
	mean (95% CI)	mean (95% CI)		mean (95% CI)	mean (95% CI)	
	n = 24*	n = 27*		n = 24*	n = 27*	
WHOQOL-BREF^†^						
Physical domain^†^ (MCID 7.7%)	68.2 (63.1-73.3)	68.0 (63.0-73.0)	.958	66.1 (60.9-71.3)	64.9 (59.9-69.8)	.721
Psychological domain^†^ (MCID 6.3%)	65.9 (60.7-71.2)	65.0 (59.9-70.2)	.796	64.0 (57.7-70.2)	59.8 (53.8-65.7)	.312
Social Relationships domain^†^ (MCID 6.4%)	66.0 (59.2-72.9)	64.2 (57.7-70.7)	.691	72.0 (62.2-81.9)	58.7 (49.6-67.7)	.044
Environment domain^†^ (MCID 5.7%)	74.8 (70.9-78.8)	74.0 (70.1-77.9)	.764	74.5 (68.5-80.5)	69.9 (64.2-75.6)	.255
Overall perception of life and health (Question 1+2)^†^ (MCID 8.8%)	65.4 (57.1-73.7)	61.6 (53.6-69.6)	.491	61.4 (53.7-69.2)	59.4 (52.2-66.6)	.686
FACT-G (MCID ≥ 6 points)						
Total score^†^	76.0 (71.5-80.5)	73.0 (68.5-77.4)	.328	72.7 (68.2-77.3)	68.7 (64.3-73.0)	.188
Physical well-being^†^	20.9 (19.2-22.7)	20.4 (18.7-22.1)	.667	19.5 (17.8-21.3)	20.7 (19.1-22.3)	.307
Social/Family well-being^†^	20.3 (18.6-22.1)	19.0 (17.3-20.6)	.244	19.2 (17.4-21.1)	16.3 (14.6-18.0)	.019
Emotional well-being^†^	17.3 (16.2-18.4)	16.8 (15.7-17.9)	.509	16.9 (15.7-18.1)	16.2 (15.1-17.3)	.373
Functional well-being^†^	17.2 (15.4-19.0)	16.2 (14.5-18.0)	.437	16.6 (15.0-18.3)	15.5 (13.9-17.0)	.296
CFS-D						
Total score^‡^	24.3 (20.8-27.8)	25.5 (22.1-28.8)	.63	25.9 (22.6-29.2)	26.5 (23.4-29.6)	.794
Physical fatigue subscale^‡^	11.1 (9.3-12.9)	11.5 (9.8-13.2)	.722	11.5 (9.8-13.2)	11.8 (10.2-13.4)	.788
Affective fatigue subscale^‡^	5.3 (4.3-6.3)	4.9 (3.9-5.9)	.565	5.7 (4.9-6.6)	5.1 (4.3-5.9)	.314
Cognitive fatigue subscale^‡^	7.8 (6.7-9.0)	8.5 (7.3-9.6)	.411	8.7 (7.4-10.0)	8.9 (7.6-10.1)	.833
PSQ						
Overall score⁺	45.3 (40.5-50.1)	45.4 (40.8-50.0)	.972	47.7 (42.7-52.7)	48.6 (43.9-53.2)	.796
Worries subscale⁺	37.7 (31.1-44.3)	42.2 (35.8-48.5)	.314	41.2 (33.3-49.1)	42.9 (35.5-50.2)	.74
Tension subscale⁺	44.6 (38.2-51.0)	47.2 (41.0-53.3)	.549	45.6 (39.8-51.3)	51.3 (45.9-56.6)	.135
Joy subscale⁺	47.1 (38.6-55.5)	51.6 (43.7-59.5)	.434	42.0 (35.0-49.0)	46.4 (40.1-52.8)	.338
Demands subscale⁺	44.4 (39.3-49.6)	44.3 (39.4-49.3)	.978	45.5 (39.8-51.1)	47.1 (41.8-52.4)	.66

Means with 95% CI adjusted for baseline value and stratification variables and *P*-values.

Abbreviations: CI, confidence interval; *P*, *P*-value for treatment effect. WHOQOL-BREF, World Health Organization Quality of Life Assessment; FACT-G, functional assessment of cancer therapy-general; CFS-D, Cancer Fatigue Scale; PSQ, Perceived Stress Questionnaire.

* Number of randomized patients; number of patients in analyses may vary, see Figure 1.

† Higher values indicate better quality of life.

‡ Lower values indicate less suffering of fatigue.

⁺ Higher values indicate high level of perceived stress

### Results of the Qualitative Study

In total, 20 patients (mean age 56.7 years (SD 12.0)) participated in the focus group interviews (MFW n = 11; MW n = 9). Of the 32 contacted patients who participated in at least 6 of 8 course dates, 12 declined participation mainly because of time constraints.

The results of the qualitative analysis showed that patients described positive effects of both study interventions, especially on well-being and an improved approach to stress management and cancer therapy-related side-effects, such as functional capacity and fatigue. However, the effects in both groups varied substantially: MFW primarily promoted mindfulness and self-care as well as acceptability in BC patients. Patients in the MFW group reported a feeling of “inner strength” and experienced an improvement in coping with their diagnoses and lasting side-effects.

“I just got a different awareness of my body [. . .], because I always integrate these mindfulness exercises into my everyday life. It helps me, because there are moments when I do not at all feel well and (I have) this fatigue [. . .] and my performance level has not come back either. [. . .] And [. . .] I can deal better with my fears, which are always there, because I just do these exercises and this meditation and that just helps me [. . .], I just feel stronger. [. . .] So even if I sometimes feel incredibly weak physically, I feel strong inside.” MW_9, MindfulWalking_II

In comparison, MW primarily activated and empowered the patients as a result of the physical exercise; the feeling of physical exhaustion, the experience of control over one’s body and its improvement in capacity through walking were emphasized by the patients.

“But it was [. . .] a positive [. . .] exhaustion, [. . .]. And over time [. . .] the condition (got) [. . .] better and better.” W_7, Walking_II

Participants of both groups stressed the importance of such interventions for BC patients after the end of primary therapy, and they were very satisfied with the study and perceived the intervention as effective.

“It's nice [. . .] that conventional medicine is now opening up a little to the subject of holism [. . .]. (I) would think, it would be great if there was something like that [. . .], that is generally offered to women during cancer [. . .] or afterward [. . .] and that it is recognized as a [. . .] prevention course by the health insurance companies [. . .].” MW_9, MindfulWalking_II

However, the results of the inductive qualitative analysis also showed feelings of ambivalence regarding study participation: engaging with the topic of the BC disease and identifying oneself as a BC patient vs. the wish to be healthy and being “my old self” again. Further concerns regarding study participation were time constraints and management. Multiple responsibilities, including those of wage labor, family, and household, which patients found themselves increasingly exposed to again after the end of primary therapy, seemed to make it difficult to set up and participate in weekly intervention appointments.

“If you work, have children at home and then [. . .] have to drive that far, [. . .] it can be, [. . .] (a) bit difficult.” W_6, Walking_II

In both groups, criticism was related to the questionnaires; on the basis of Likert scales and the items asked, personal changes and feelings were not sufficiently captured. Additionally, the duration of the study intervention could have been even longer; if participants had missed individual appointments due to time constraints and time management issues, 8 or fewer dates would have been too few.

The detailed presentation of comprehensive qualitative results will be provided in a separate paper.

## Discussion

Both study interventions were positively evaluated by patients and showed positive pre-post effects in disease-specific health-related outcomes, especially regarding quality of life, stress reduction and alleviation of fatigue. The quantitative analysis revealed only minor differences between both groups after 8 and 16 weeks without statistical significance. Thus, no additional effects of the combination of mindfulness meditation techniques plus walking practices could be demonstrated compared to MW alone based on the quantitative data. In contrast, the qualitative data elucidated the characteristics of both study interventions more clearly and possible underlying mechanisms of action: MFW encouraged a conscious, mindful, and self-caring approach to oneself, whereas MW strengthened a feeling of self-efficacy and empowerment through physical activity.

To our knowledge, the present study is the first randomized controlled trial exploring the effects of an MFW intervention compared to MW alone in patients with BC. Since there was already some evidence of the effects of walking and mindfulness interventions on BC patients, we decided against a comparison to a routine care group. Due to the comparison of 2 active groups with each other, it was possible to explicitly examine the additional effects of the mindfulness component. The explorative study design and mixed-methods approach allowed us to understand individual experiences and feasibility as well as possible effects of both interventions in a broad, multidimensional manner to draw conclusions for further confirmative studies.

Further strengths of this study include its pragmatic real-world approach and an interdisciplinary stakeholder meeting held at the conception phase of the study. Here, the need for further offerings of Integrative Medicine supporting BC patients after the end of primary therapy was emphasized; thus, an important inclusion criterion for study participation was determined. Additionally, the wide range of outcome parameters to explore possible effects of the intervention within this pilot study were determined.

However, there are also limitations to the study. Due to the design of the study, blinding of participants was not possible. Since all outcome parameters were assessed independently and self-perceptively, impacts of patients’ expectations and assumptions of the allocated intervention could not be completely ruled out, even though expectations of the study intervention were assessed at baseline prior to randomization and were comparable between groups. The lack of a third study group with routine care alone does not allow to estimate the unspecific effects of MFW with respect to MW. Furthermore, patient recruitment proved to be more difficult than expected; with 51 patients included in the study, the number of participants in the study was not only too small to detect differences between to active groups but also imbalanced in some of the baseline characteristics. Additionally, the drop-out rate was relatively high. This might be explained by the results of the qualitative data. On one hand, the qualitative data confirmed patients’ wish for Integrative Medicine and further support in the course of disease. Especially after the end of primary therapy, patients claimed to have felt left alone with their situation and feelings by their social environment and medical practitioners. On the other hand, patients described a feeling of ambivalence toward study participation: a wish to not identify oneself as a BC patient anymore but to put it behind them and be healthy again. These results are comparable to van Lankveld et al,^58^ who discussed recruitment problems in psychosocial oncology research. Furthermore, time constraints and management after the end of primary therapy was another concern and reason for non-participation. Thus, it should be considered whether feasibility would improve if the interventions were offered within 6 months after the primary therapy.

The patients’ motivation, high satisfaction with MFW/MW and general assessment of the effectiveness of MFW/MW after intervention contradicted the high drop-out rate and difficulties in recruitment. Qualitative data and reported reasons for drop out and non-participation showed that time constraints and management were the main obstacles in study participation, which is comparable to that reported in Jeitler et al.^59^ To improve recruitment and adherence rate of the intervention, an earlier intervention time within 6 months after the primary treatment needs to be considered for further confirmatory studies.

While no severe AEs could be detected in this study, a higher number of AEs was documented. The individual impact of the documented AEs for the patients remained unclear. To assess the safety of the interventions, it is necessary to carefully evaluate the AEs that occurred, as most were training-related and therefore predictable. They have to be differentiated from the occurrence of panic attacks in MFW, which can be more problematic and require further investigation.

In this study, disease-specific quality of life, measured with the FACT-G total score, improved in both study groups, with no significant group differences after 8 and 16 weeks. However, with respect to a minimal clinically important difference of 6 points in FACT-G, the findings were clinically relevant for MFW after 8 and 16 weeks but not for MW. This might indicate that MFW may have more sustainable effects than MW; however, confirmatory trials with a greater patient population and long-term follow-up are needed to prove this hypothesis.

The pre-post effects in this study of 5% to 10% were comparable to the extent of pre-post effects in a study by Jeitler et al^59^ Results of this prospective cohort study showed that the combination of mind-body-medicine and lifestyle modifications within the context of day care clinic treatment over 12 weeks compared to a waiting list group can contribute to a clinically relevant and statistically significant improvement of quality of life in cancer patients as measured by FACT-G.

The findings of this study support the idea that MFW may reduce stress in BC patients, especially with respect to the subdomains *worries* and *tension*. However, the effects of MFW were not superior to MW after 8 and 16 weeks. Differences between groups became clearer by the qualitative data: MFW promotes the ability to conquer self-perceived stress in everyday life through mindfulness meditation techniques, especially breathing techniques, as well as the ability to say no and therefore promote a self-caring approach to oneself and one’s own resources. In contrast, MW seems to be a more direct and immediate tool for stress management by the physical component. A stress reducing effect could be found in randomized controlled trials and meta-analysis for MBSR interventions compared to usual care or “no MBSR” control conditions in cancer patients.^25-27,30^ Comparing a 4-week mindful walking intervention to a waiting list, Teut et al^33^ showed large positive effects of mindful walking in healthy individuals with elevated subjectively perceived stress levels in a randomized controlled trial. However, in comparison to our study, the effects shown by Teut et al might have been larger because the patients were psychologically distressed individuals without a chronic disease, such as BC, and the control group was a mere waiting group without an active study intervention.

Cancer-related fatigue measured by the CFS-D improved in a clinically relevant area based on a MCID of 3 to 6 points (5%-10%). Like Spahn et al,^60^ who evaluated the effects of a multimodal mind-body program including physical activity compared to a walking intervention alone on chronic fatigue symptoms in BC patients, we did not find differences between groups in the quantitative data. In contrast, the qualitative data from this mixed-methods study indicated possible differences between the interventions: while MFW promoted acceptability and improvement in handling long term disease- and therapy-related consequences, such as fatigue, MW more directly activated patients and improved self-perceived physical capacity.

In the interpretation of the quantitative results, it needs to be considered that, since participants of MFW showed a higher home practice frequency, this may have influenced effects toward MFW.

The qualitative results from this mixed-methods study coincided with quantitative outcomes with respect to the perceived effectiveness and the beneficial pre-post effects in both groups. The qualitative data, however, further illustrated possible differences between the interventions. This emphasizes the benefits of the mixed-methods approach and the triangulation of quantitative and qualitative data in this pilot study as well as for future confirmative randomized trials.^61^

In summary, the following conclusions can be drawn from this pilot study for future confirmatory studies: a larger sample size and a comparison to usual care and mindfulness alone should be included in the study; an earlier intervention time within 6 month after the primary treatment needs to be considered to improve recruitment and adherence; the questionnaires on quality of life (WHOQOL-BREF and FACT-G), stressfulness (PSQ), and fatigue (CFS-D) showed the greatest pre-post effects in this pilot study and appear to be the most suitable; the triangulation of quantitative and qualitative data proved to be useful in order to investigate possible effects in their entirety.

## Conclusions

Results of this randomized controlled pilot trial with a nested qualitative study component indicated the importance of such offers in the after primary oncologic care setting. Both study interventions might have positive pre-post effects in BC survivors without significant differences between groups. Qualitative results revealed that both interventions seem to address different resources: selfcare and acceptability in MFW versus empowerment and self-efficacy in MW. These important findings should be further investigated in future confirmative mixed-methods studies.

## Supplemental Material

sj-doc-1-ict-10.1177_15347354211066067 – Supplemental material for Feasibility and Possible Effects of Mindful Walking and Moderate Walking in Breast Cancer Survivors: A Randomized Controlled Pilot Study With a Nested Qualitative Study Part Click here for additional data file. Supplemental material, sj-doc-1-ict-10.1177_15347354211066067 for Feasibility and Possible Effects of Mindful Walking and Moderate Walking in Breast Cancer Survivors: A Randomized Controlled Pilot Study With a Nested Qualitative Study Part by Maren Luise Schröder, Barbara Stöckigt, Sylvia Binting, Tatjana Tissen-Diabaté, Nikola Bangemann, Ute Goerling, Matthias Kröz, Jens-Uwe Blohmer, Miriam Ortiz and Benno Brinkhaus in Integrative Cancer Therapies

sj-docx-1-ict-10.1177_15347354211066067 – Supplemental material for Feasibility and Possible Effects of Mindful Walking and Moderate Walking in Breast Cancer Survivors: A Randomized Controlled Pilot Study With a Nested Qualitative Study Part Click here for additional data file. Supplemental material, sj-docx-1-ict-10.1177_15347354211066067 for Feasibility and Possible Effects of Mindful Walking and Moderate Walking in Breast Cancer Survivors: A Randomized Controlled Pilot Study With a Nested Qualitative Study Part by Maren Luise Schröder, Barbara Stöckigt, Sylvia Binting, Tatjana Tissen-Diabaté, Nikola Bangemann, Ute Goerling, Matthias Kröz, Jens-Uwe Blohmer, Miriam Ortiz and Benno Brinkhaus in Integrative Cancer Therapies

sj-docx-2-ict-10.1177_15347354211066067 – Supplemental material for Feasibility and Possible Effects of Mindful Walking and Moderate Walking in Breast Cancer Survivors: A Randomized Controlled Pilot Study With a Nested Qualitative Study Part Click here for additional data file. Supplemental material, sj-docx-2-ict-10.1177_15347354211066067 for Feasibility and Possible Effects of Mindful Walking and Moderate Walking in Breast Cancer Survivors: A Randomized Controlled Pilot Study With a Nested Qualitative Study Part by Maren Luise Schröder, Barbara Stöckigt, Sylvia Binting, Tatjana Tissen-Diabaté, Nikola Bangemann, Ute Goerling, Matthias Kröz, Jens-Uwe Blohmer, Miriam Ortiz and Benno Brinkhaus in Integrative Cancer Therapies
