# Supplementary material for: Feasibility and Possible Effects of Mindful Walking and Moderate Walking in Breast Cancer Survivors: A Randomized Controlled Pilot Study With a Nested Qualitative Study Part
Source: Integr Cancer Ther. 2022 Jan 19;21:15347354211066067. doi: 10.1177/15347354211066067 (PMC8777370; doi:10.1177/15347354211066067)
Supplement: sj-doc-1-ict-10.1177_15347354211066067 – Supplemental material for Feasibility and Possible Effects of Mindful Walking and Moderate Walking in Breast Cancer Survivors: A Randomized Controlled Pilot Study With a Nested Qualitative Study Part [file sj-doc-1-ict-10.1177_15347354211066067.doc]

**Supplementary File 1** Outcome parameters at 4 weeks. Means with 95% CI; adjusted for baseline value and stratification variables and p-values

|  | **4 weeks** | | |
| --- | --- | --- | --- |
| **Outcomes** | **Mindful Walking**  **mean (95% CI)**  **n=24*** | **Walking**  **mean (95% CI)**  **n=27*** | **p-value** |
| **WHOQOL-BREF^†^**  **Physical domain^†^ (MCID 7,7%)**  **Psychological domain^†^ (MCID 6,3%)**  **Social Relationships domain^†^ (MCID 6,4%)**  **Environment domain^†^ (MCID 5,7%)**  **Overall perception of life and health (Question 1+2)^†^ (MCID 8,8%)** | 67.2 (62.9 - 71.5)  61.5 (56.9 - 66.1)  63.7 (58.3 - 69.1)  74.9 (71.3 - 78.6)  58.3 (51.2 - 65.3) | 66.0 (61.8 - 70.2)  59.3 (54.9 - 63.8)  63.4 (58.2 - 68.5)  71.6 (68.1 - 75.2)  55.3 (48.4 - 62.2) | 0.68  0.483  0.925  0.186  0.538 |
| **FACT-G (MCID ≥ 6 points)**  **Total score^†^**  **Physical well-being^†^**  **Social/Family well-being^†^**  **Emotional well-being^†^**  **Functional well-being^†^** | 73.2 (70.0 - 76.4)  21.1 (19.7 - 22.6)  19.4 (17.7 - 21.0)  16.2 (15.0 - 17.3)  16.5 (15.5 - 17.5) | 69.8 (66.6 - 73.0)  20.2 (18.8 - 21.7)  17.6 (16.0 - 19.3)  15.8 (14.6 - 16.9)  16.1 (15.1 - 17.1) | 0.129  0.361  0.139  0.605  0.581 |
| **PSQ**  **Overall score⁺**  **Worries subscale⁺**  **Tension subscale⁺**  **Joy subscale⁺**  **Demands subscale⁺** | 44.7 (40.1 - 49.4)  37.7 (31.1 - 44.3)  45.4 (39.5 - 51.4)  49.3 (42.7 - 55.9)  43.6 (38.0 - 49.1) | 49.6 (45.3 - 54.0)  46.8 (40.4 - 53.1)  53.4 (47.7 - 59.1)  47.4 (41.2 - 53.5)  45.2 (39.8 - 50.6) | 0.121  0.047  0.054  0.671  0.667 |
| **HADS**  **Total score′**  **Anxiety subscale′**  **Depression subscale′** | 14.6 (13.1 - 16.1)  8.0 (7.1 - 8.9)  6.5 (5.5 - 7.6) | 15.5 (14.1 - 17.0)  8.4 (7.6 - 9.3)  7.0 (6.0 - 8.0) | 0.356  0.45  0.519 |
| **ASKU**″ | 3.7 (3.5 - 3.9) | 3.7 (3.5 - 3.9) | 0.895 |
| **FFA^**^** | 36.6 (34.0 - 39.3) | 34.5 (32.0 - 37.0) | 0.227 |
| **Pain (NRS) ^##^**   - **because of cancer** - **because of other disease** | 2.8 (2.1 - 3.5)  4.0 (2.9 - 5.1) | 3.1 (2.5 - 3.8)  4.3 (3.2 - 5.4) | 0.45  0.693 |

CI = confidence interval, p = p-value for treatment effect, *Number of randomized patients; number of patients in analyses may vary, see Fig. 1. WHOQOL-BREF = World Health Organization Quality of Life Assessment, FACT-G = Functional Assessment of Cancer Therapy-General, PSQ = Perceived Stress Questionnaire, HADS = Hospital Anxiety and Depression Scale, ASKU = General self-efficacy short scale, FFA = Freiburg Mindfulness Inventory, NRS = numeric rating scale. ^†^higher values indicate better quality of life; ⁺higher values indicate high level of perceived stress; ′higher values indicate higher severity of symptoms; ″higher values indicate better self-efficacy; **higher values indicate higher mindfulness; ^##^lower values indicate less suffering of pain
