# Supplementary material for: Feasibility and Possible Effects of Mindful Walking and Moderate Walking in Breast Cancer Survivors: A Randomized Controlled Pilot Study With a Nested Qualitative Study Part
Source: Integr Cancer Ther. 2022 Jan 19;21:15347354211066067. doi: 10.1177/15347354211066067 (PMC8777370; doi:10.1177/15347354211066067)
Supplement: sj-docx-2-ict-10.1177_15347354211066067 – Supplemental material for Feasibility and Possible Effects of Mindful Walking and Moderate Walking in Breast Cancer Survivors: A Randomized Controlled Pilot Study With a Nested Qualitative Study Part [file sj-docx-2-ict-10.1177_15347354211066067.docx]

**Supplementary File 2** Further outcome parameters at 8 and 16 weeks. Means with 95% CI; adjusted for baseline value and stratification variables and p-values

|  | **8 weeks** | | | **16 weeks** | | |
| --- | --- | --- | --- | --- | --- | --- |
| **Outcomes** | **Mindful walking**  **mean (95% CI)**  **n=24*** | **Walking**  **mean (95% CI)**  **n=27*** | **p-value** | **Mindful walking**  **mean (95% CI)**  **n=24*** | **Walking**  **mean (95% CI)**  **n=27*** | **p-value** |
| **HADS**  **Total score′**  **Anxiety subscale′**  **Depression subscale′** | 13.9 (12.2 - 15.6)  7.6 (6.6 - 8.6)  6.3 (5.1 - 7.5) | 13.6 (12.0 - 15.3)  7.8 (6.8 - 8.7)  5.8 (4.6 - 6.9) | 0.808  0.793  0.507 | 14.3 (12.0 - 16.6)  7.7 (6.5 - 8.9)  6.6 (5.2 - 8.0) | 14.9 (12.8 - 17.0)  8.6 (7.5 - 9.7)  6.3 (5.0 - 7.6) | 0.688  0.253  0.705 |
| **Trait autonomic regulation^#^**  **Autonomic regulation total score^#^**  **Orthostatic-circulative aR subscale^#^**  **Rest-/activity aR subscale^#^**  **Digestive aR subscale^#^** | 39.3 (37.6 - 41.0)  15.6 (14.5 - 16.7)  16.5 (15.3 - 17.6)  7.2 (6.7 - 7.6) | 38.9 (37.2 - 40.5)  15.6 (14.6 - 16.7)  16.1 (15.0 - 17.2)  7.2 (6.7 - 7.7) | 0.722  0.912  0.618  0.856 | 39.4 (37.7 - 41.1)  16.1 (15.2 - 17.1)  16.1 (15.0 - 17.2)  7.2 (6.6 - 7.7) | 38.8 (37.2 - 40.4)  15.6 (14.7 - 16.5)  16.2 (15.2 - 17.3)  7.1 (6.6 - 7.6) | 0.593  0.398  0.888  0.904 |
| **ASKU″** | 4.0 (3.8 - 4.2) | 3.8 (3.6 - 4.0) | 0.19 | 3.9 (3.6 - 4.2) | 3.7 (3.5 - 4.0) | 0.28 |
| **FFA**** | 37.4 (35.2 - 39.6) | 37.0 (34.9 - 39.1) | 0.77 | 37.0 (34.6 - 39.4) | 35.1 (32.9 - 37.3) | 0.217 |
| **Pain (NRS) ^##^**   - **because of cancer** - **because of other disease** | 1.8 (1.0 - 2.5)  3.6 (2.5 - 4.7) | 2.4 (1.7 - 3.2)  4.1 (3.0 - 5.2) | 0.217  0.509 | 2.2 (1.4 - 2.9)  3.7 (2.7 - 4.8) | 2.4 (1.7 - 3.0)  4.1 (3.1 - 5.1) | 0.718  0.617 |

CI = confidence interval, p = p-value for treatment effect, *Number of randomized patients; number of patients in analyses may vary, see Fig. 1. HADS = Hospital Anxiety and Depression Scale, ASKU = General self-efficacy short scale, FFA = Freiburg Mindfulness Inventory, aR = autonomic regulation, NRS = numeric rating scale. ′higher values indicate higher severity of symptoms; **^#^**lower values indicate less autonomic regulation; ″higher values indicate better self-efficacy; **higher values indicate higher mindfulness; ^##^lower values indicate less suffering of pain
